# Aged Chinese-origin rhesus macaques infected with SIV develop marked viremia in absence of clinical disease, inflammation or cognitive impairment

**DOI:** 10.1186/s12977-018-0400-y

**Published:** 2018-02-01

**Authors:** Stephanie J. Bissel, Kate Gurnsey, Hank P. Jedema, Nicholas F. Smith, Guoji Wang, Charles W. Bradberry, Clayton A. Wiley

**Affiliations:** 1 University of Pittsburgh, 3550 Terrace Street, S758 Scaife Hall, Pittsburgh, PA 15261 USA; 2 Veterans Affairs Pittsburgh Healthcare System, 4100 Allequippa Street, Pittsburgh, PA 15213 USA; 3 Present Address: National Institute on Drug Abuse, 251 Bayview Boulevard, Baltimore, MD 21224 USA

**Keywords:** HIV, Simian immunodeficiency virus, HIV-associated neurocognitive disorder, Cognition, Aging, Rhesus macaque

## Abstract

**Background:**

Damage to the central nervous system during HIV infection can lead to variable neurobehavioral dysfunction termed HIV-associated neurocognitive disorders (HAND). There is no clear consensus regarding the neuropathological or cellular basis of HAND. We sought to study the potential contribution of aging to the pathogenesis of HAND. Aged (range = 14.7–24.8 year) rhesus macaques of Chinese origin (RM-Ch) (n = 23) were trained to perform cognitive tasks. Macaques were then divided into four groups to assess the impact of SIVmac251 infection (n = 12) and combined antiretroviral therapy (CART) (5 infected; 5 mock-infected) on the execution of these tasks.

**Results:**

Aged SIV-infected RM-Ch demonstrated significant plasma viremia and modest CSF viral loads but showed few clinical signs, no elevations of systemic temperature, and no changes in activity levels, platelet counts or weight. Concentrations of biomarkers of acute and chronic inflammation such as soluble CD14, CXCL10, IL-6 and TNF-α are known to be elevated following SIV infection of young adult macaques of several species, but concentrations of these biomarkers did not shift after SIV infection in aged RM-Ch and remained similar to mock-infected macaques. Neither acute nor chronic SIV infection or CART had a significant impact on accuracy, speed or percent completion in a sensorimotor test.

**Conclusions:**

Viremia in the absence of a chronic elevated inflammatory response seen in some aged RM-Ch is reminiscent of SIV infection in natural disease resistant hosts. The absence of cognitive impairment during SIV infection in aged RM-Ch might be in part attributed to diminishment of some facets of the immunological response. Additional study encompassing species and age differences is necessary to substantiate this hypothesis.

**Electronic supplementary material:**

The online version of this article (10.1186/s12977-018-0400-y) contains supplementary material, which is available to authorized users.

## Background

Combined antiretroviral therapy (CART) has completely or almost completely suppressed HIV replication translating to significant reductions in mortality and morbidity in infected individuals. Along with better long-term outcomes and decreased frequency of AIDS-related illness among HIV-infected patients taking suppressive CART, deaths attributable to HIV infection have decreased by 48% since the peak in 2005 [1]. Nevertheless, non-AIDS defining illnesses including neurocognitive deficits normally associated with aging are often observed in the HIV-infected population.

The term HIV-Associated Neurocognitive Disorders (HAND) is used to describe a spectrum of clinical disorders ranging from asymptomatic neurocognitive impairment to mild neurocognitive disorder to HIV-associated dementia (HAD), the clinical correlate of HIV encephalitis [2]. Although CART has been found to prevent or delay these neurocognitive sequelae, less severe neurocognitive dysfunction remains a common comorbidity. Anywhere from 25 to 50% of the HIV-infected population on successful long-term CART experience mild-moderate HAND [3–9] and as this population ages, they are two to seven times more likely to have mild cognitive impairment than their seronegative peers [10–14]. Other comorbidities and behavioral traits have been reported to be risk factors for HAND such as cardiovascular-related conditions [15, 16], obesity [17], diabetes [16, 17], hyperlipidemia [16], tobacco use [16], hepatitis C co-infection [18], alcohol and substance abuse [14, 19], education [20, 21], poverty [21], sleep disorders [14], and psychiatric comorbidities [14]. Many of these conditions are typically associated with aging and contribute to the assumption that HIV-infected individuals undergo a premature aging process. This theory has much traction, but it has been questioned whether available data support the theory of accelerated aging [22]. Regardless, the risk of developing HAND is likely confounded by many of these variables and will be challenging to tease apart, especially with older age.

We know little about the pathogenesis of HAND and why it persists in the presence of CART [23]. Before CART was introduced, HAD was associated with increased HIV RNA in the cerebrospinal fluid (CSF) in patients with severe immunosuppression, arguing for a direct effect due to viral replication in the central nervous system (CNS) [24–27]. However, in populations with access to CART, there is no strong correlation between HIV RNA in the CSF and neurocognitive impairment [28, 29]. Together, these observations suggest that a constellation of immune and viral processes contributes to cognitive dysfunction. Potential mechanisms include comorbidities, “hit and run” effect of HIV entering the CNS early and causing long term neurodegenerative damage [30–34], chronic inflammation in the periphery and/or CNS, substance abuse, age and CART neurotoxicity.

HIV-infected individuals experience complications associated with age earlier than non-infected individuals [35]. Treated HIV-infected individuals experience chronic inflammation, hypercoagulation, and an increased risk of non-AIDS-related morbidity and mortality [36]. There are few preclinical models that can address the cellular and system bases of age-related neurocognitive dysfunction during HIV infection. Since non-human primate (NHP) brains are highly concordant with cortical and subcortical architecture of humans, they can be employed to study neurological abnormalities and neuropathogenesis in conjunction with aging. Human and NHP have different but comparable lifespans where the effects of aging on complex immune and nervous system function can be studied. Thus, SIV infection of NHPs offers a valid model to study the effects of aging and chronic lentiviral infection.

To model the less severe forms of HAND, we trained aged Chinese-origin rhesus macaques (RM-Ch) to perform cognitive tests and then assigned them to four performance-matched experimental groups. Half of the RM-Ch were then infected with SIVmac251. Cognitive function along with clinical and virological assessments were followed for 8 months, at which point half of the infected and half of the mock-infected RM-Ch were administered CART for an additional 6 months. Since RM-Ch are reported to have lower viremic peaks and set points, greater maintenance of CD4 T cell counts, and significantly longer survival times than rhesus macaques of Indian origin [37–39], we anticipated a slow disease progression that could recapitulate neurological abnormalities observed during HIV infection.

## Methods

### Subjects

Aged (13.5–23.5 year at beginning of study) female rhesus macaques of Chinese origin (*n* = 23) (RM-Ch) with no previous behavioral training were used for the present study. Macaques were housed and maintained according to American Association of Laboratory Animal Care standards. The University of Pittsburgh’s Institutional Animal Care and Use Committee approved all experimentation. Following acquisition, animals were habituated to pole and collar handling and placement in a behavioral primate chair (Primate Products, Immokalee, FL). Collars were fitted with compact accelerometers (Actical, Philips Respironics, Murrysville, PA) to detect sleep and activity patterns. Temperature sensor monitors (DST micro-T temperature logger, Star-Oddi, Iceland) were implanted in the mid-scapular region. To learn to accept water reinforcement rewards, subjects were trained to use a sipper tube attached to the chair. Water was regulated 7 days/week and supplemented (weekly average of 20 ml/kg/d) at the end of each day after training and testing and over the weekend.

For antibody response determination, additional plasma from 9 young adult rhesus macaques (3–11 year old) infected with SIVmac251 for a median of 153 days post-infection was used. Five of these macaques were classified as controllers of infection, while four macaques were classified as progressors.

### Water reinforcement rewards

During cognitive testing, water rewards were given to reinforce positive responses to stimuli. Animals with > 20% weight loss from commencement of water regulation were removed from water restriction until weights rebounded to acceptable levels before continuing water regulation. During the study period, nine animals (5 SIV-infected; 4 mock-infected) required temporary removal from water restriction lasting from 18 to 89 days in duration. These animals were not dehydrated or losing weight because of SIV infection, rather the animals were overweight when water regulation was initiated. Four SIV-infected macaques were also removed from water restriction due to illness prior to euthanasia for SIV-related (n = 1) or other (n = 3) reasons. Cognitive assessment data was not obtainable during these periods.

### Cognitive assessments

Cognitive assessments took place in a sound attenuated chamber (model AB4240, Eckel Industries, Cambridge, MA) fitted with a 40 W light and white-noise generator. The E-prime software suite (Psychology Software Tools, Sharpsburg, PA) coupled with a CarrollTouch infrared touch screen (Elo Touch Solutions, Milpitas, CA) was used for all stimulus presentation, response recording, and data processing. Baseline measures for cognitive tasks were evaluated at the end of the training period to establish performance and age-matched experimental groups using a grade assessment statistic as indicated in Table 1. Cognitive assessments were conducted Monday through Thursday.

**Table 1:** Study groups, clinical outcomes, neuropathological and systemic pathological findings, and SIV infection in brain regions and systemic organs

Group	Primate #	Age (years)	# days infected	Completed study	Periods off study^a^	Grade/assessment statistic	Neuropathological findings	Systemic pathological findings	mf ctx	cau/put/cc	insula/bg	thal, hip	cb	occ ctx
SIV + CART−	205	22.8	442	Y	Y	A	Corticospinal tract microglial activation		–	–	–	–	–	–
	221	15.9	442	Y	N	A+		Bronchopneumonia, mild; nephritis, mild	–	–	–	–	–	–
	211	20.9	124	N	Y	B	SIV encephalomyelitis		+ 3.5 × 10^5^	+	+	+	+	+
	202	21.4	253	N	Y	C−	Bacterial meningitis; rare SIV + cell in spinal cord		− 5.2 × 10^1^	–	–	–	–	–
	209	22.3	275	N	Y	C−			–	–	–	–	–	–
	214	23.2	175	N	N	A−	CMV radiculitis; CMV meningoencephalitis; diffuse microglial activation WM > GM	CMV aspiration pneumonia	− 1.4 × 10^0^	–	–	–	–	–
	220	20.5	442	Y	Y	F−			–	–	–	–	–	–
SIV-CART-	212	21.7	NA	Y	Y	A+		Nephritis, mild, focal						
	222	17.0	NA	Y	N	A+								
	204	22.0	NA	Y	Y	C		Medullary fibrosis						
	203	25.0	NA	Y	N	C−								
	216	18.5	NA	N	Y	D+	Global cortical contracted eosinophilic neurons	Splenic angiosarcoma; kidney angiosarcoma & hemorrhagic cyst; prominent macrophages in mes LNs, small bowel, & colon lamina propria						
	218	18.1	NA	N	N	B+		Benign liver cyst; pancreatic islet cells have cleared cytoplasm; inflamed coronary plaque; chronic inflammation of fallopian tubes; type II fiber atrophy in quadriceps muscles; interstitial inflammation in left kidney; metaplastic tubules in right kidney						
SIV-CART+	213	26.0	NA	Y	N	A+								
	217	18.8	NA	Y	N	B		Diffuse myocardial fibrosis, mild						
	210	19.8	NA	Y	Y	C	Secondary demyelination in lateral column							
	200	21.8	NA	Y	N	D+								
	219	18.7	NA	Y	N	D+		Papillary muscle infarction, chronic						
SIV + CART+	207	19.8	442	Y	N	A+		Chronic bronchitis, low level; macrophage infiltration of cardiac muscle, very mild	–	–	–	–	–	–
	201	18.6	342	N	Y	B	SIV meningoencephalitis, mild; SIV + microglial nodules; SIV myelitis; vacuolar myelitis	Consolidating pneumonia (non-SIV-related), severe	+ 8.7 × 10^2^	+		+	+	+
	208	19.7	441	Y	N	C+	Menigitis, mild, unknown etiology		− 3.7 × 10^−1^	–	–	–	–	–
	215	18.7	442	Y	Y	D+	PVCI	Alveolar macrophage infiltration, moderate, pigmented	–	R	–	–	–	–
	224	18.1	441	Y	Y	F	Cerebellar infarct, chronic	Chronic inflammation of striated muscle adventitia	–	–	–	–	–	–

Group	Spleen	Liver	Spinal cord	Thymus	mes LN	Lung	Small bowel	Colon	Heart	Ovary	ax LN	Quad muscle	Kidney	Other
SIV + CART−	+	–	–	–	+	–	–	+	–	–	+	–	+	
	+	R	–	–	–	–	–	–	–	–	R	–	–	
	+	R	+	–	+	+	+	+	R	–	+	–	+	
	+	–	R	R	+	–	+	+	–	–	+	–	+	
	+	–	–	R	+	–	+	+	–	–	+	–	–	Peritracheal LN, adrenal gland
	+	–	–	+	+	+	+	+	–	+	+	–		Peripheral nerves
	–	–	–	–	–	–	+	–	–	–	–	–	–	
SIV-CART−														
SIV-CART+														
SIV + CART+	–	–	–	–	–	–	–	–	–	–	–	–	–	
	+	R	+	–	+	–	+	+	–	+	+	–	–	
	+	–	R	–	–	–	–	+	–	–	+	–	–	
	+	–	–	–	–	–	+	–	–		–	–	–	
	–	–	–	–	–	–	+	–	–	–	+	–	–	

Study groups were selected on basis of age and performance. Systemic and neuropathological findings are summarized for each animal. In situ hybridization for SIV RNA was perfromed in each area listed. Quantitation of SIV RNA in the midfrontal cortex was performed by RTPCR. Positive values are shown as copies/μg RNA

*CMV* cytomegalovirus, *WM* white matter, *GM* gray matter, *PVCI* perivascular chronic inflammation, *mf* midfrontal, *ctx* cortex, *cau* caudate, *put* putamen, *cc* corpus callosum, *bg* basal ganglia, *thal* thalamus, *hip* hippocampus, *cb* cerebellum, *occ* occipital, *mes* mesenteric, *LN* lymph node, *ax* axial, *quad* quadriceps, *musc* muscle

^a^Periods off study for weight loss, illness or quarantine for false positive TB test

### Speeded motor task

On each cognitive assessment day, a stimulus was presented on a touchscreen to start each trial of a 200 trial session. After pressing and holding the stimulus, the trial was advanced to presentation of a new stimulus. Attending correctly to the new stimulus resulted in a water reward and removal of the stimulus (scored a correct response). No water reward was offered upon an incorrect response and the stimulus was removed. Reward levels were amplified with speed of response. Accuracy, response time and percent completion were recorded. Eight animals did not acquire the ability to hold the stimulus to advance the trial, so their task was modified. For these animals, each trial began with presentation of the second stimulus, and a successful touch of that stimulus was scored as accurate, with the response time determined as the duration between presentation of the stimulus and an accurate touch. Analyses were binned by every 2 weeks post-infection (wpi).

### Plasma and CSF draws

Plasma and CSF draws were performed/attempted prior to SIV inoculation and every 2 wpi. Samples were drawn at the conclusion of the week’s cognitive tasks to provide for recovery before tasks were resumed. Samples were aliquoted and stored at − 80 °C.

### SIV inoculation

Macaques were inoculated with SIVmac251 (obtained from the Vaccine and Prevention Research Program, Division of AIDS, National Institute of Allergy and Infectious Diseases and Quality Biological Inc., Gaithersburg, MD from Dr. Ronald Desrosiers) by intravenous injection at 0 wpi. Macaques were observed daily for clinical signs of anorexia, weight loss, lethargy, or diarrhea. When deemed necessary by an examining veterinarian, animals with poor health were euthanized before completing the study. Due to age-related conditions such as congestive heart failure, kidney disease, and obstructive blood clots, euthanasia was necessary in both SIV-infected (5/12 animals) and mock-infected macaques (2/11 animals).

### Antiretroviral therapy

Beginning 38 wpi, animals in the antiretroviral treatment groups received daily subcutaneous injections of reverse transcriptase inhibitors bis{[(isopropoxycarbonyl)oxy]methyl}({[(2*R*)-1-(6-amino-9*H*-purin-9-yl)-2 propanyl]oxy}methyl)phosphonate (TDF,Tenofovir disoproxil; 5.1 mg/kg) and 4-amino-5-fluoro-1-[(2*R*,5*S*)-2-(hydroxymethyl)-1,3-oxathiolan-5-yl]-1,2-dihydropyrimidin-2-one (FTC, emtricitabine, 50 mg/kg). Animals in no treatment groups received saline injections. TDF and FTC were generously provided by Gilead Sciences, Inc. (Foster City, CA) through Material Transfer Agreements.

### Quantitation of SIV RNA in plasma and CSF

Virions from 1 ml of plasma or 200–500μl of CSF were pelleted by centrifugation at 23,586×*g* for 1 h, 4 °C. Total RNA was extracted from the pellet using Trizol reagent (Invitrogen, ThermoFisher Scientific, Waltham, MA). A standard quantitative RT-PCR was performed with 10μl RNA for each sample based on amplification of conserved sequences in *gag* [40].

### Tissue collection and processing

At the conclusion of the study, animals were euthanized and perfused with phosphate buffered saline. Brain, spinal cord, spleen, liver, thymus, mesenteric and axial lymph nodes, lung, small bowel, colon, heart, ovary, quadriceps muscles, and kidney were collected. Portions of each tissue were fixed in 10% buffered formalin and paraffin embedded. After making coronal sections (~ 5 mm), every other section of the right brain hemisphere was fixed, while remaining sections were snap frozen in ~ 100 mg pieces of midfrontal cortex, caudate, putamen, hippocampus and cerebellum.

### Quantitation of SIV RNA in brain tissue

Approximately 100 mg midfrontal cortex was disrupted in Trizol reagent using a Mini BeadBeater (Glen Mills, Inc., Clifton, NJ) to isolate RNA. SIV gag copy numbers were determined as described previously [40, 41] except the total amount of RNA was used to normalize the samples. Total RNA was quantitated using Quant-iT Ribogreen RNA Assay Kit (Invitrogen, ThermoFisher Scientific).

### Histological assessment

Formalin fixed paraffin embedded sections from brain and other organs were processed for hematoxylin and eosin staining and CD68 and GFAP immunohistochemistry as described before [42]. To assess distribution and abundance of SIV infected cells, in situ hybridization (ISH) was performed as previously described [43] using riboprobes targeting portions of *gag*, *pol*, and *env* of the molecular clone of SIVmacBK28 [44].

### Flow cytometry

After overnight shipment, 100 ml aliquots of EDTA-anticoagulated whole blood were incubated with a mastermix of fluorochrome-conjugated antibodies against a lymphocyte panel or a monocyte panel. The lymphocyte panel included antibodies against the following surface molecules: CD20 (L27, AlexaFluor 700), CD45 (D058-1283, PerCP), CD4 (SK3, PE-Cy7), CD8 (SK1, AmCyan), CD3 (SP34-2, PE-CF594). The monocyte panel included antibodies against the following surface molecules: HLA-DR (L243, APC-Cy7), CD45 (D058-1283, PerCP), CD3 (SP34-2, PE-CF594), CD20 (L27, AlexaFluor 700), CD14 (M5E2, FITC), CD16 (3G8, PacificBlue). All antibodies were from BD Biosciences (San Jose, CA). After lysing red blood cells, samples were acquired on a BD LSRII at Immunology Services unit of the Wisconsin National Primate Research Center (University of Wisconsin, Madison, WI). Data analysis was performed using FlowJo version 9.6.2 (Tree Star, Inc., Ashland, OR).

### sCD14 ELISA

Plasma soluble CD14 concentrations were measured in duplicate using the Human CD14 Quantikine ELISA kit (R&D Systems, Minneapolis, MN) according to the manufacturer’s protocol.

### Multiplex analysis of plasma inflammation markers

ProcartaPlex Multiplex Immunoassays (Affymetrix eBioscience, San Diego, CA) were used to detect non-human primate IL-6, IL-18, CXCL10 and TNF-α at the following time points: baseline (0 wpi), acute infection (2 and 4 wpi), and prior to initiation of CART (34/36 wpi). The 4 wpi time point was not measured in mock –infected macaques. Samples were read by the University of Pittsburgh Cancer Institute LUMINEX Facility using the Luminex 100 reader (Luminex Corporation, Austin, TX).

### Humoral responses

ELISA analyses of the humoral immune responses to SIV envelope protein were tested at baseline, ~ 24 wpi, and necropsy as previously described [45] with modifications. A reference plasma with strong anti-Env antibody concentrations was aliquoted and stored at − 80 °C. A batch of EIA/RIA high binding plates were coated overnight with 0.08 μg/ml of rgp130 SIV mac251 (ImmunoDx, Woburn, MA) in PBS (pH 7.4) using 100 μl/well at 4 °C. Plates were blocked with 200 μl B3T buffer (150 mM NaCl, 50 mM Tris–HCl, 1 mM EDTA, 3.3% fetal bovine serum, 2% bovine serum albumin, 0.07% Tween 20) for 1 h at 37 °C. An aliquot of the reference plasma and test plasmas were serially diluted in B3T buffer and added to the plate in duplicate at 100 μl/well for 1 h at 37 °C. 100 μl of horseradish peroxidase-conjugated goat anti-monkey IgG (Rockland Immunochemicals, Inc., Limerick, PA) at 1:10,000 was added for 1 h at 37 °C. Plates were washed 6× with 0.1% Tween 20 in PBS after each step then developed using SureBlue TMB 1-Component Microwell Peroxidase Substrate (SeraCare Life Sciences, Milford, MA) for approximately 25 min. A TMB Stop Solution (SeraCare) was added and plates were read at 450 nm. The OD values for the reference plasma were used to interpolate relative values of anti-Env antibody concentrations using Prism 7 software (GraphPad Software, Inc.).

### Statistical analysis

Mann–Whitney tests were used to compare mock-infected controls with SIV-infected macaques. For time course comparisons to baseline values, Kruskal–Wallis tests followed by Dunn’s multiple comparison tests were performed. Statistical analyses were performed with Prism 6 (GraphPad Software, San Diego, CA).

## Results

### Modeling HAND in aged SIV-infected macaques

**Fig. 1:**
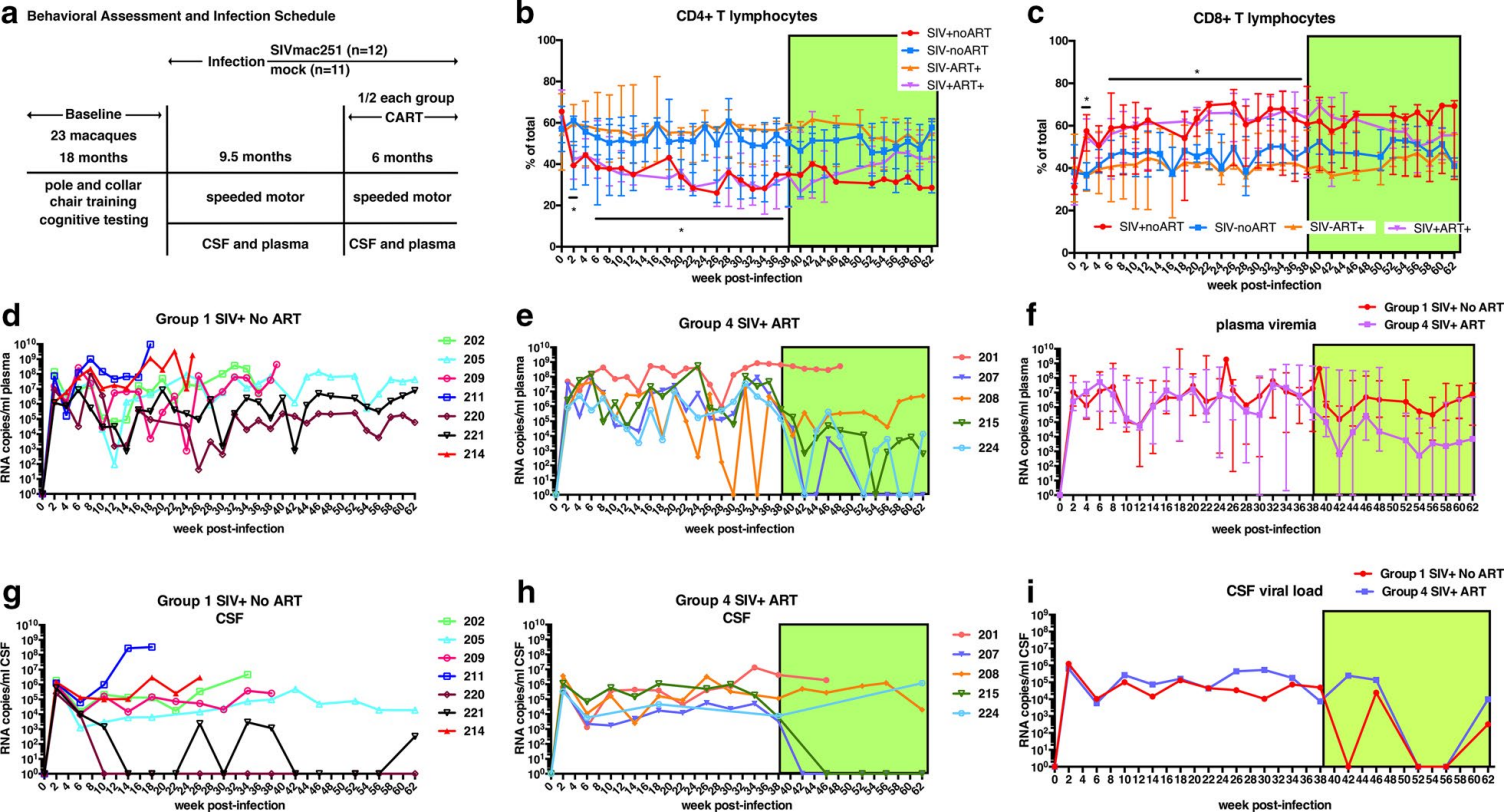
SIV-infection of aged macaques of Chinese origin has significant viremia. Timeline of cognitive testing, infection and CART (**a**). The proportion of CD4+ lymphocytes declines slightly in aged macaques of Chinese origin but recovers after CART. Longitudinal median ± standard error of peripheral blood proportions for CD4+ lymphocytes (**b**) and CD8+ lymphocytes (**c**) in SIV-infected and mock-infected aged RM-Ch. Plasma (**d**) and CSF (**g**) SIV viral loads from 7 SIV-infected macaques that did not receive treatment show significant viral replication. Plasma (**e**) and CSF (**h**) SIV viral loads from 5 SIV-infected macaques that received CART at 38 wpi show decreased viral replication during the treatment period. Median plasma (**f**) and mean CSF (**i**) SIV viral load of SIV-infected macaques that received CART compared to macaques that did not. The bars in (f) represent the upper and lower values. Asterisks indicate *P* < 0.05 for indicated time points. Kruskal–Wallis tests were used for (**b**), (**c**), (**f**), and (**i**). The green shaded area represents the period macaques received CART

A group of 23 aged macaques (age range at time of infection = 14.7–24.8 year; median age = 18.6 year) underwent a cognitive training period of 18 months to acclimate and teach the following: pole and collar handling, placement in a behavioral primate chair, water rewards, and cognitive tasks. Initially, it was planned to train the animals for a sensorimotor speeded response task and two executive function tasks. However, within the 18-month training period, this was not possible due to difficulties in training aged animals that had never undergone behavioral training (subjects were retired former breeders). Subjects began training with the speeded motor task, which was continued for the remainder of the study. Behavioral performance was assessed as detailed in Fig. 1a.

Using baseline measurements for the speeded motor task (grade assessment statistic), four performance and aged-matched groups were established at the end of the training period (Table 1): SIV-infected, no treatment (initially n = 6); mock-infected, no treatment (initially n = 5); mock-infected CART treated (initially n = 6); and SIV-infected, CART treated (initially n = 6). Aged adults were difficult to train to interact with the touchscreens. Training techniques utilized in the past with younger animals, such as target training then bridging into a new behavior were often unsuccessful with this cohort. Eight aged RM-Ch did not reach the final stage of training for the final task at the end of the training period, so the task was modified accordingly for these animals. For comparison with past younger animals, the number of training sessions to learn to interact with the touchscreen ranged from 18 to 90 sessions for the aged RM-Ch, while 100% of a group of 14 young adult males successfully learned the task in half the time (11–46 sessions).

### Acute RM-Ch infected with SIV were clinically asymptomatic

Macaques were infected with SIVmac251 at the end of the 18-month training period. Similar to individuals with acute HIV infection, infection of macaques with SIV sometimes results in an acute febrile response accompanied by development of a maculopapular rash, lymphadenopathy, diarrhea, weight loss, transient platelet decrease, and changes in sleep and motor activity [38, 46–51]. Surprisingly, the aged SIV-infected RM-Ch exhibited minimal to no clinical signs of infection; however, some other studies have observed that RM-Ch exhibit fewer clinical signs than RM of Indian origin [37]. Acute changes in weight from baseline after SIV-inoculation were similar to mock-infected animals for the first 2 wpi (Additional file 1: Fig. S1). At 3 and 4 wpi, SIV-infected animals showed greater weight loss than mock-infected RM-Ch, but since the variation in weight changes observed pre-infection was frequent and of similar amplitude, this change could not be attributed to SIV infection. There was no change in body temperature or platelet counts (Additional file 1: Figs. S2 and S3), and no lymphadenopathy was palpable. Comparison of temperature to plasma and CSF viral loads for individual SIV-infected animals are shown in Additional file 1: Fig. S6. Finally, activity counts and sleep patterns were similar to pre-infection levels and between the SIV-infected and mock-infected animals (data not shown).

### Aged RM-Ch infected with SIV showed significant viremia

Despite absence of clinical signs during acute infection, aged SIV-infected RM-Ch had a median plasma viral load at 2 wpi of 9.65 × 10^6^ copies/ml (range, 5.88 × 10^5^–1.43 × 10^8^ copies/ml) (Fig. 1). The viral load remained elevated in animals that did not receive CART through the remainder of the experiment with median viral loads at 10^7^ copies/ml at several time points; however, some macaques exhibited significant variation over the course of infection. CART lowered median plasma viral load 2–3 logs during the 6-month treatment period (Fig. 1f). CSF viral load had an acute peak at 2 wpi (median, 1.23 × 10^6^ copies/ml; range, 2.54 × 10^5^–3.67 × 10^6^ copies/ml) (Fig. 1i). Two SIV-infected macaques had undetectable to low levels of CSF virus after acute infection (Fig. 1g), while the remaining SIV-infected animals maintained CSF viral loads that ranged from 10^3^ to 10^6^ copies/ml. Three of the eight SIV-infected macaques that died before the end of the study showed elevated CSF viral load. Two of these animals demonstrated SIV encephalitis (#211; necropsy CSF viral load = 3.3 × 10^8^ copies/ml) or SIV meningoencephalitis (#201; necropsy CSF viral load = 1.9 × 10^6^ copies/ml), while the other macaque had a bacterial meningitis with infrequent SIV-infected cells in the spinal cord but not in the brain (#202; necropsy CSF viral load = 4.68 × 10^6^ copies/ml). In macaques that received CART, detection of viral RNA in the CSF was completely eliminated in two macaques, decreased one log in one macaque, and showed a small decrease in one macaque that died a few weeks following CART initiation. One macaque showed increased viral RNA in the CSF with CART, but since few samples could be collected for this animal, effectiveness of CART throughout the treatment period was unknown.

### Changes in peripheral blood cell populations in aged RM-Ch infected with SIV

The proportion of CD4+ T cells in SIV-infected macaques dropped from a median of 62–41% of T cells at 2 wpi, with a reciprocal increase in proportion of CD8+ T cells (Fig. 1a, b). This proportion decrease remained steady throughout the length of infection, although during the treatment period, animals receiving CART began to show T cell proportions similar to baseline and non-infected macaques. Absolute CD4+ T cell counts also decreased after infection, but the decrease was only significant at 32 and 34 wpi. Total CD8+ T cell counts were transiently increased during acute infection and at various time points thereafter (*P* < 0.05 at 22, 26, 40 and 52 wpi). NK cell counts followed a similar transient increase pattern (*P* < 0.05 at 26 and 52 wpi). Total cell counts for CD4+ and CD8+ T cells, NK cells and especially B cells showed transient fluctuations in the non-infected groups as well. T and NK cell counts tended to increase in non-infected macaques, while B cell counts decreased over time for all groups (Additional file 1: Fig. S4). Comparison of CD4+ T cell and CD8+ T-cell counts to plasma and CSF viral loads for individual SIV-infected animals are shown in Additional file 1: Fig. S7. Monocyte subset populations were also followed to examine expansion of inflammatory monocytes (Additional file 1: Fig. S5). The first few measurements after infection were not readable, so baseline cell counts of monocyte subsets were not available. There was little difference in median cell counts of classical monocyte populations (CD14 + CD16−). Intermediate or inflammatory monocytes (CD14 + CD16 +) showed transient increases at 12, 22, and 26 wpi, but the proportion of these cells was similar in SIV-infected and non-infected macaques.

### Effect of age on survival and CNS infection

Five of the twelve (41%) aged SIV-infected macaques required euthanasia prior to the conclusion of the experiment, while two of the eleven (18%) non-infected macaques did not finish the study (Table 1). The SIV-infected macaques that did not complete the study had a higher median plasma viremia and CSF viral load at euthanasia than the SIV-infected macaques who completed the study (at the last time point prior to treatment, plasma: 5.3 × 10^8^ copies/ml vs 1.82 × 10^7^ copies/ml; CSF: 1.9 × 10^6^ copies/ml vs 3.80 × 10^2^ copies/ml). SIV-infection could be attributed as the reason for euthanasia in one macaque that developed SIV pneumonitis and encephalomyelitis. The other infected macaques succumbed to obstructed blood flow to the bowel, pneumonia, rhesus cytomegalovirus infection and bacterial meningitis. Using *in situ* hybridization, SIV infected cells were detected in the brains of three SIV-infected macaques at necropsy. Two macaques (#201 and #211) had infected lesions in every region examined, while one had infrequent infected cells in the caudate and putamen (#215) (Table 1). An additional two macaques (#202 and #208) also showed infrequent infected cells in the spinal cord. SIV RNA was detected in midfrontal cortex of four of the SIV-infected macaques that required euthanasia prior to the conclusion of the experiment and one that finished the study (median, 51.6 copies/μg RNA; range, 0.4–3.5 × 10^5^ copies/μg RNA) (Table 1). Other neuropathological findings included mild to moderate deep white matter microgliosis, corticospinal tract degeneration, and chronic infarcts, but these findings were not exclusively associated with infection status. In the periphery, SIV-infected cells were detected in several organs of the non-treated SIV-infected macaques. While SIV infected cells were detected in treated SIV-infected macaques, the frequency and range was less than the non-treated macaques.

### Neither SIV infection nor CART elicited changes in aged RM-Ch sensorimotor behavioral testing outcome

**Fig. 2:**
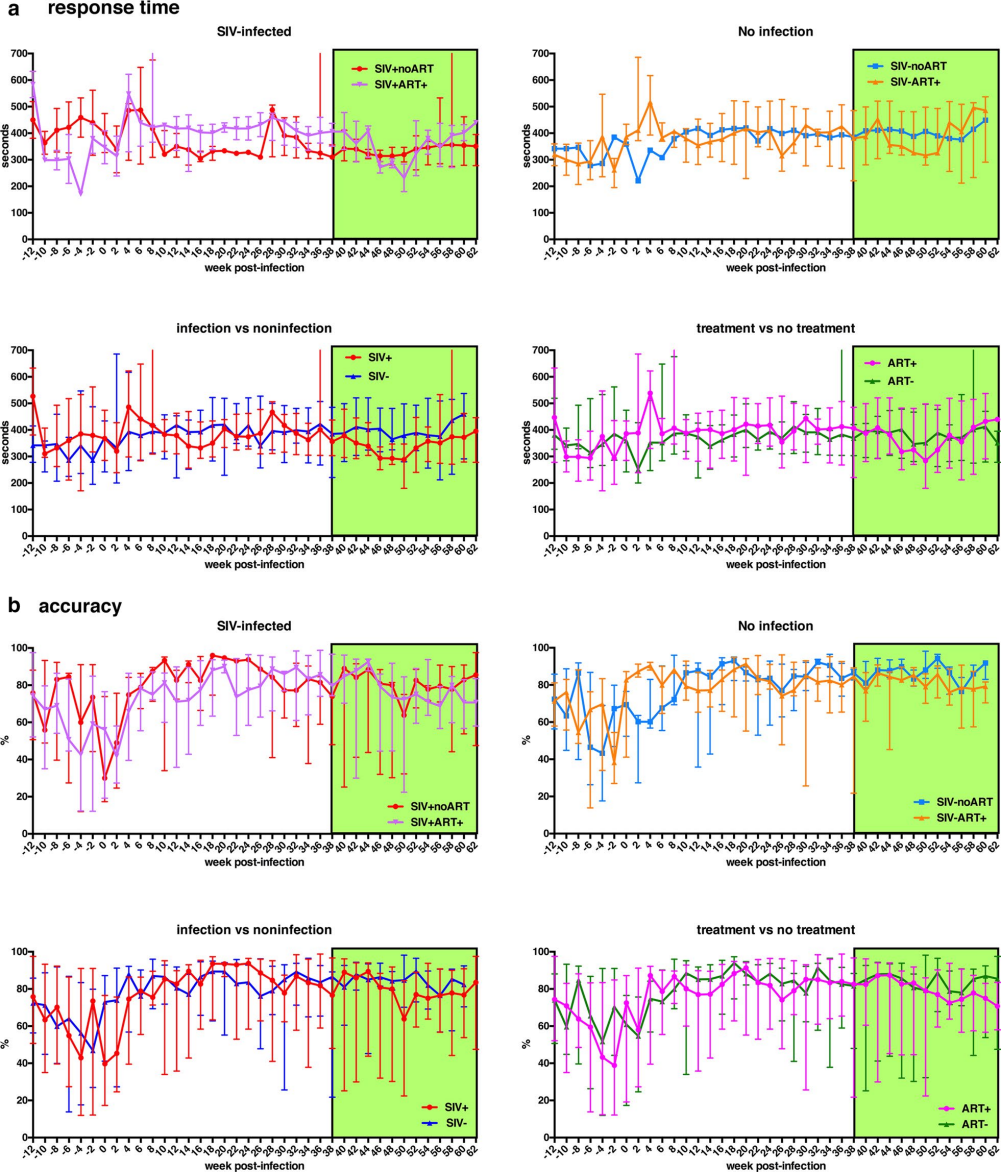
Neither infection or CART impacted performance on a speeded motor task in aged Chinese macaques. Comparison of median response times and accuracy did not show significant differences. Shown here are between group comparisons of mean ± standard error speeded motor performance response time (**a**) and accuracy (**b**). Analyses for response time and accuracy were binned by every 2 wpi over the course of training period, SIVmac251 or mock infection and CART. Differences between SIV-infection without CART versus SIV-infection with CART (SIV-Infected), Mock-infection without CART versus Mock-infection with CART (No Infection), SIV-infected versus Mock-Infected (Infection vs Noninfection), and CART versus PBS (treatment vs no treatment) are shown for a and b. Kruskal–Wallis tests were used to compare results displayed in each graph, but no statistically significant differences were found. The green shaded area represents the period macaques received CART

Baseline performances of each macaque were assigned a grade assessment statistic to create performance and age-matched groups. In these aged RM-Ch, there was a range of performance ability that did not correlate with age of the animal or any other observed variable. The median response time and percent accuracy in the speeded motor test was similar in SIV-infected and non-infected groups in the 12 weeks prior to infection (Fig. 2). During the acute infection period, both SIV-infected and non-infected groups showed improvements in response time and percent accuracy. This was attributed to the aged macaques slowly continuing to acquire proficiency/skill in the given task. After approximately 10 wpi, the response time and percent accuracy began to plateau. Neither infection or treatment had a significant effect on response time or percent accuracy (Fig. 2). Comparison of reaction time and accuracy to plasma and CSF viral loads for individual SIV-infected animals are shown in Additional file 1: Fig. S8, while the reaction time and accuracy of individual mock-infected macaques are shown in Additional file 1: Fig. S9. These results suggest that aged RM-Ch are capable of learning and improving tasks even during acute SIV-infection.

### SIV-infection of aged RM-Ch was characterized by minimal inflammation

**Fig. 3:**
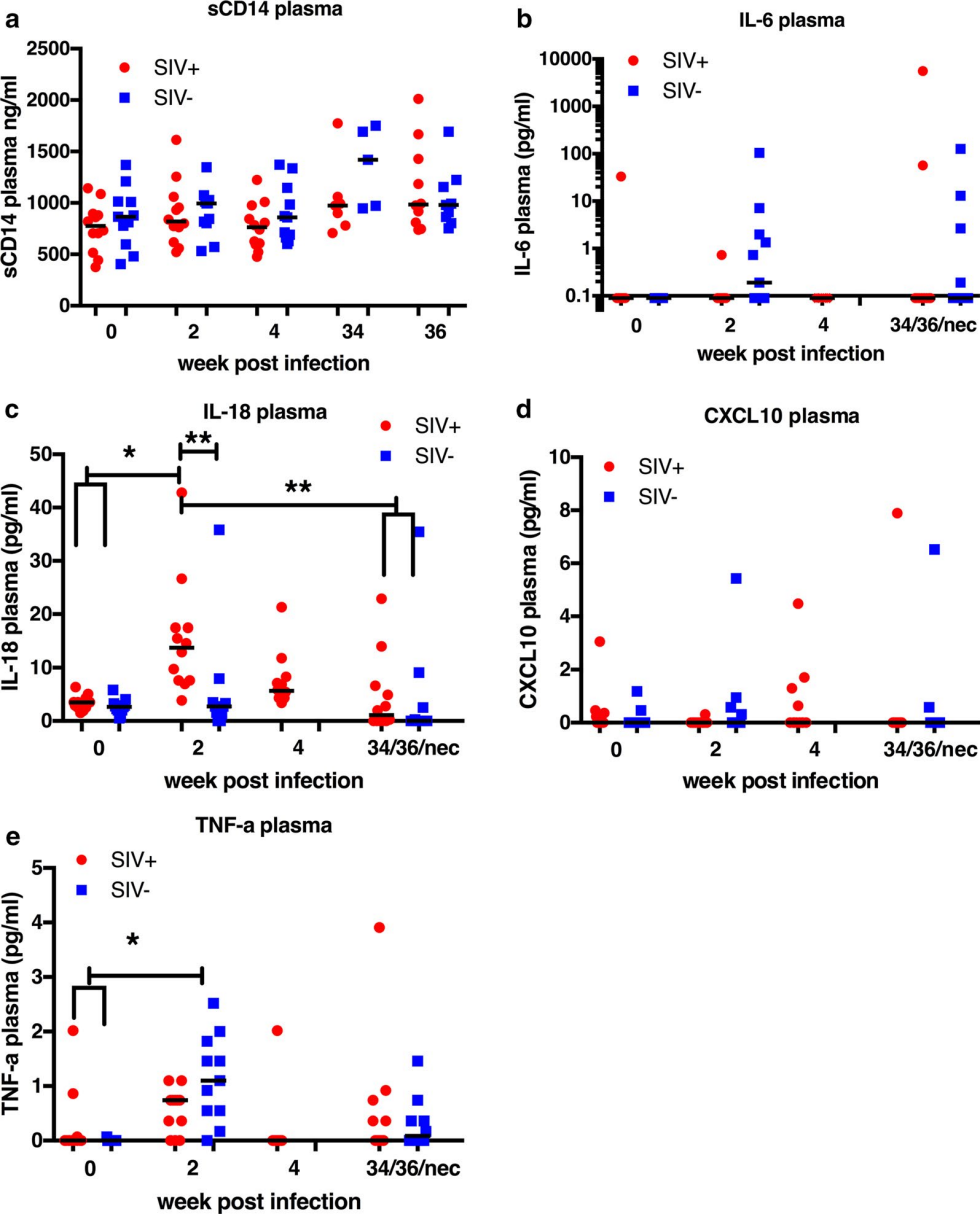
No elevation of hallmarks of chronic inflammation during lentiviral infection in aged SIV-infected Chinese macaques. IL-18 is elevated in aged SIV-infected macaques of Chinese origin during acute infection, but other hallmarks of chronic inflammation during lentiviral infection remain stable. Median plasma concentrations of soluble CD14 (sCD14) (**a**), IL-6 (**b**), IL-18 (**c**), CXCL10 (**d**), and TNF-α (**e**) at 0, 2, 4 wpi and 34 or 36 wpi or necropsy (34/36/nec). 0 wpi represents baseline, 2 and 4 wpi represent acute infection and 34/36/nec represent chronic infection. Kruskal–Wallis tests were used to analyze differences between groups. *P < 0.05. **P < 0.01

Pathogenic HIV/SIV infection is characterized by acute and persistent inflammation. To assess whether aged SIV-infected RM-Ch showed indications of a typical inflammatory response during infection, plasma sCD14, IL-6, IL-18, CXCL10 and TNF-α concentrations were assessed during the acute time points and prior to initiation of therapy. sCD14 levels were similar in both SIV-infected and non-infected macaques at all time points examined (Fig. 3a). IL-6, CXCL10 and TNF-α concentrations were undetectable in many of the animals regardless of infection status. However, IL-18 was significantly increased in SIV-infected macaques at 2 wpi (Fig. 3c). Overall, these results suggest that aged macaques of Chinese origin do not respond to SIV infection in the inflammatory manner characteristic of younger macaques and patients with HIV infection [52–57].

**Fig. 4:**
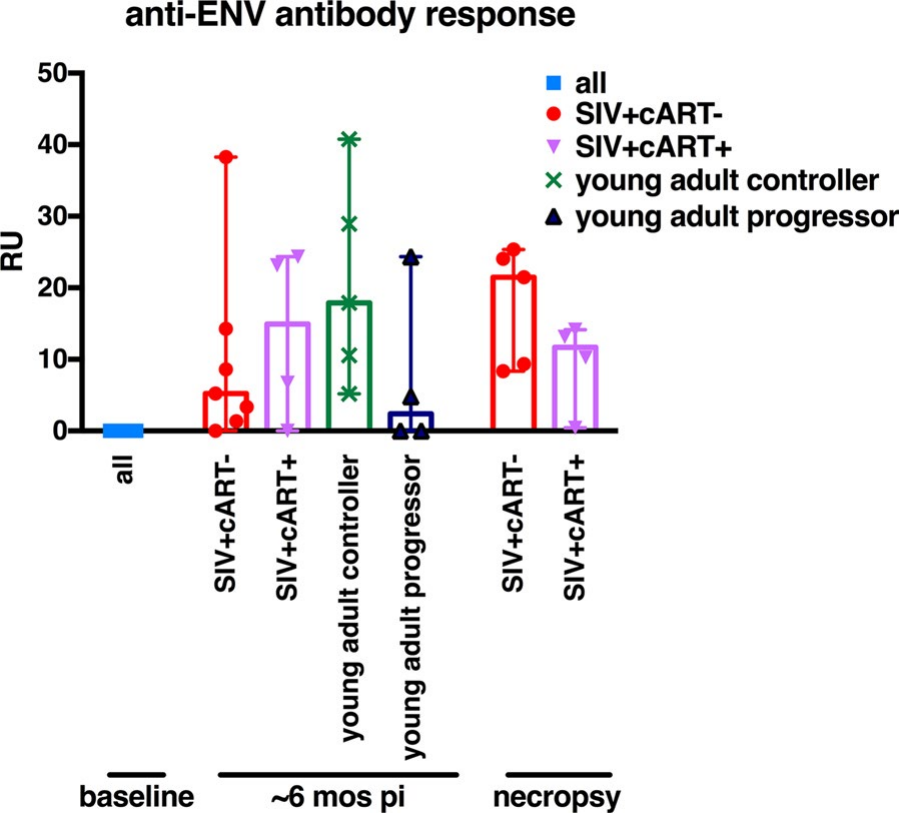
Aged SIV-infected Chinese macaques generate antibody responses similar to progressors and less than controllers. Median Gp130 Env antibody response determined by ELISA. Kruskal–Wallis tests were used to compare each time point

To address an aspect of the adaptive immune response in SIV-infected aged RM-Ch, we compared anti-Env antibody responses to SIV-infected young RM of Indian origin. It has been reported that RM-Ch generate stronger antibody responses them RM of Indian origin [55]. The majority of SIV-infected aged macaques generated detectable anti-Env antibody responses (Fig. 4). Two aged SIV-infected RM-Ch (#211 and #201) that required euthanasia prior to completion of the study failed to generate detectable anti-Env antibodies similar to two young adult progressor macaques. Three of four of the young adult animals with disease progression generated minimal if any anti-Env responses. Two of the remaining three aged SIV-infected macaques that required euthanasia prior to the conclusion of the experiment also showed minimal anti-Env antibody responses, while the animal that succumbed to obstructed blood flow to the bowel developed substantial anti-Env antibody responses. The aged SIV-infected macaques that completed the study generated variable anti-Env antibody responses similar to the young adult controller macaques.

## Discussion

To understand processes contributing to HAND and aging, our objective was to model HAND in aged NHP in order to dissect the pathological and eventually mechanistic basis of this range of neurocognitive disorders. SIV infection of aged RM-Ch was quite different than reported for young adults [37, 38, 51, 57–72]. They showed minimal to no clinical signs upon infection, with no elevations of systemic temperature and no changes in activity levels, platelet counts or weight. Yet, the aged macaques demonstrated significant plasma viremia and modest CSF viral loads. Neither acute nor chronic SIV infection nor CART had a significant impact on accuracy, speed or percent completion in a speeded motor test. Since this study did not include young adult RM-Ch, we have used historic data that were not generated in the same conditions for comparisons. Although this does not detract from the findings, it will be important to perform additional study encompassing species and age differences to substantiate our conclusions.

### Modeling human age-related neurological degeneration

Our study plan was to obtain macaques with brains similar in age to 50-year-old humans that could be trained for cognitive assessment and evaluated during chronic infection. Reasoning on the basis of proportional chronology, we estimated the 14–20-year-old macaques used in this study were roughly analogous to 50–60-year-old humans. It has been reported that macaques over 20 years of age show neuropathological changes of ageing analogous to those seen in humans over 60 years of age [73]. In our experience, only the most aged of nonhuman primates (~ 30 years old) have shown amyloid beta accumulation (a hallmark of pathological aging in humans commencing at 60–65 years of age but observed at younger ages in early onset neurodegenerative disease).

As different macaque species have variable susceptibility to SIV disease progression, it was important to choose a macaque species that was resilient to rapid disease progression and thus permit long-term study. We reasoned this would enable the animals to survive the training and testing paradigm and mitigate conditions that would confound the behavioral studies. The RM-Ch subspecies best fit these requirements.

We had several difficulties modeling the current commonly described forms of HAND in aging individuals beginning with training the 14–20-year-old macaques to learn cognitive tasks. Compared to past young adults, the aged macaques were recalcitrant to training, and despite increasing the training period, the macaques were only able to reliably perform the speeded motor task. Then after SIV infection, the aged RM-Ch failed to show clinical signs and we could not discriminate differences in neurocognitive performance. Despite matching groups for baseline cognitive task performance, the heterogeneous responses of the small groups of outbred aged RM-Ch further limited the ability to sensitively discern cognitive impairment. This highlights the difficulty and limitations of experimentally modeling the issues facing aviremic HIV-infected patients on effective long-term CART, yet experiencing HAND, in aged macaques. Might a different experimental approach or model be more suitable? This will have to be interrogated systematically in order to develop a system to address how chronic inflammation impacts cognition in the face of effective viral suppression.

### Aged SIV-infected RM-Ch showed marked levels of plasma viremia

Juvenile and adult RM-Ch are reported to have innate resistance to SIV infection compared to rhesus macaques of Indian origin [37, 38, 58]. Levels of viral replication in RM-Ch tend to be lower than in macaques of Indian origin, but significant inter-individual variation in disease progression has been reported [59, 60]. Surveying the literature for plasma viremia in younger SIV-infected RM-Ch, peak viremia ranges from 10^3^ to 10^8^ copies/ml with an approximate mean of 5 × 10^7^ copies/ml, while set point viremia ranges from 10^3^ to 10^7^ copies/ml [37, 38, 51, 57, 59–72]. Although viral strain and route of inoculation influence direct comparison of viral loads, the median plasma viremia of the aged RM-Ch in this study was in line with these published values at 9.65 × 10^6^ copies/ml; however, the set point viremia was maintained at a median of 10^7^ copies/ml, which is similar to the high end of the reported set point range.

### Aged RM-Ch showed minimal clinical signs of SIV infection

Despite this viral load, little clinical evidence of infection was apparent. Some investigators have made similar observations with young RM-Ch showing fewer clinical signs of infection than RM of Indian origin and little appreciable weight loss [37]. However, others have observed that approximately half of young RM-Ch present with lymphadenopathy and experience weight loss, wasting, and diarrhea [38, 51]. Weight in the SIV-infected aged RM-Ch was similar to non-infected macaques. Temperature also remained remarkably stable, even during acute infection. This is contrary to infection of younger rhesus macaques that demonstrate hyperthermia during acute infection that lasts approximately 3 months [50], though these RM were most likely of Indian origin. Declines in platelet counts are reported to be an indicator of disease progression in HIV and SIV infection [49, 74], yet the aged macaques in this study did not show any alterations in platelet counts. Activity and sleep disturbances are also associated with SIV infection [50, 75, 76], but activity counts during day and night periods were similar to pre-infection and mock-infected animals. The absence of change in these clinical parameters suggests that SIV infection of aged RM-Ch is more analogous to SIV infection in the natural host (e.g. sooty mangabeys and African green monkeys), potentially for the same reason that some aspects of the immunological response to SIV such as type I IFN expression is less robust in the natural host than observed in other macaques [77]. Yet, this is a complex hypothesis to test, especially with the considerable variability observed in clinical parameters and immune activation of SIV infection of young RM-Ch [38, 51, 57, 66, 77].

Supporting the hypothesis that aged RM-Ch are refractory to clinical SIV-related disease, CD14 + CD16 + monocyte subset proportions and counts were similar in infected and non-infected macaques throughout the course of infection suggesting absence of an inflammatory environment that promotes this phenotype. Monocyte subsets are known to undergo dynamic changes as a function of duration of HIV/SIV infection and are variably reported to correlate or not with development of SIV encephalitis or HAND [78–82]. However, SIV-infected aged RM-Ch did show a decrease in the proportion of CD4+ T cells during acute infection. This population remained decreased throughout the length of infection (or until treatment). The absolute median CD4+ T cell count was also decreased but was not observed in every animal and was variable. There is no consensus reported for loss of CD4+ T cells in young adult RM-Ch. While a few reports observe stable CD4 counts [62, 68] or transient CD4 loss [70], several others detect significant CD4+ T cell loss [51, 59, 60, 71, 72].

### Aged RM-Ch did not show significant sensorimotor deficits with SIV infection or CART

Despite our extensive experience training young adult rhesus macaques to perform a variety of complex neurobehavioral tasks, aged RM-Ch proved recalcitrant to training. Nevertheless, we were successful in training aged macaques for a speeded motor task where we could reliably assess their reaction time, touch screen accuracy and percent completion. Others have shown that young adult SIV-infected macaques show neurological abnormalities that can be documented through a variety of behavioral and neurophysiological tests. Motor skills, discrimination learning, discrimination retention, recognition, recency memory and attention impairments are observed early and during the chronic phase of SIV infection in adult rhesus macaques [83–88]. Some performance impairments were characterized by gradual deteriorations throughout the course of infection, while others showed sharp declines. In comparison, the aged RM-Ch did not show any significant changes on response time or percent accuracy during a speeded motor task. Why did SIV infection fail to induce neurological abnormalities in aged RM-Ch? Without the ability to test multiple realms of cognition, it is impossible to rule out deficits in other types of memory, e.g. executive function. As an alternative hypothesis, HAND could be a consequence of chronic inflammation, with the absence of sensorimotor impairment in aged RM-Ch being a reflection of the diminished clinical and immunological response.

### Neuropathology of SIV infection and CART in aged RM-Ch

Neuropathological examination did not show any overt signs of neurodegeneration in SIV-infected or mock-infected aged macaques (Table 1). Remarkably, little fluctuation in the cognitive task was observed from baseline to chronic infection. This was also true in animals with CNS-related pathology; however, these animals typically required respite from cognitive testing shortly before succumbing to an illness. While SIV RNA in the midfrontal cortex was detectable in only a few animals, the CSF SIV RNA load remained moderate in the majority of animals, so there were presumably low levels of viral replication in the CNS. While non-quantitative neuropathological assessment did not demonstrate age-related differences between infected and mock-infected aged RM-Ch, more sensitive quantitative pathological and gene expression assessments are planned.

### Virological responses of aged RM-Ch to CART

In SIV-infected animals, administration of TDF and FTC for 6 months was effective at decreasing plasma and CSF viral load in most animals. More interesting, treatment had neither a discernable positive or negative effect on neurobehavioral performance tasks for either infected or mock-infected animals. This is consistent with some human studies that have not documented neurobehavioral impairments in patients treated with CART [89]. However, it has been postulated that CART regimens potentially contribute to neurocognitive deficits by reducing dendritic arborization [90, 91].

### SIV infection in aged RM-Ch shows lack of inflammatory environment

The lack of clinical signs and cognitive impairment drove us to examine the inflammatory response in aged SIV-infected Ch-RM. A hallmark of HIV and SIV infection is chronic inflammation and activated coagulation. This increased proinflammatory state is thought to drive alterations and senescence in immune cell populations [92] and increase availability of infectable cells [93]. Increased levels of D-dimer, IL-6, sCD14, CXCL10, TNF-α, IL-18, and CCL2 among others have been shown to be increased during acute infection or chronically increased throughout infection [52–54]. Many of these inflammatory markers are also increased in aged individuals, and it has been hypothesized that HIV infection accelerates aging [36, 94]. We did not discern any elevation in these markers during infection compared to baseline measurements and mock-infected aged RM-Ch. Plasma IL-18 (an IL-1 superfamily protein produced by activated macrophages) was elevated in SIV-infected RM-Ch during acute infection, but returned to baseline during the chronic phase of infection.

Interrogation of anti-Env antibody responses showed SIV infection was not inherently immunologically silent. Most of the young adult SIV-infected progressors generated minimal to no detectable anti-Env IgG suggesting antibody responses play a role in disease progression characteristics. Overall, the aged Ch-RM generated antibody responses similar to progressors or less than young adult controllers. SIV-neutralizing antibody titers were not determined, so it is unclear whether the binding IgG detected by ELISA was functional. A comprehensive investigation of innate and adaptive immune responses during the course of SIV infection in aged Ch-RM and controls deserves further exploration to examine potential causes for the lack of clinical signs.

The overall lack of an overt inflammatory response during either acute or chronic infection is similar to that observed during nonpathogenic SIV infections in their natural hosts [95–97]. Both natural hosts and macaques respond to SIV infection with strong upregulation of type I interferon-stimulated genes (ISG) [98]. Whereas ISG levels in natural hosts are quickly restored to baseline, upregulation of ISGs become chronic in younger Ch-RM. Although ISG expression was not assessed here, no increase in CXCL10 was observed suggesting lack of sustained ISG response in aged Ch-RM.

Another potential reason for the diminished immune response in aged RM-Ch could be immune senescence. Aged macaques have been reported to show characteristics of immune senescence with increased proinflammatory status and altered immune cell populations [92]. In fact, we have noted that aged macaques have variable, delayed, and significantly weaker anti-beta-amyloid IgG levels in response to beta-amyloid immunization [73].

An elevated inflammatory milieu, such as increased sCD14 and sCD163 along with low CD4 T-cell count nadirs are also reported to predict development of HAND [99–103]. It could be hypothesized that absence of robust inflammation during acute infection, absence of many clinical signs of infection or disease and lack of chronic inflammation in the face of substantive viral replication obviate neurological damage. Although these observations warrant verification with controls demonstrating cognitive impairment concurrently with inflammation, our data are consistent with the hypothesis that HAND may be related to a chronic immune response to infection rather than the infection itself.

## Conclusions

We show that aged RM-Ch present with minimal clinical signs during SIV infection despite substantial viremia. Along with absence of indicators of disease, aged SIV-infected RM-Ch do not display deficits in cognitive tests and do not demonstrate chronic inflammation. SIV infection of aged RM-Ch did not bring about histological signs of neurodegeneration. Although these conclusions will need to be substantiated encompassing species and age differences, the observations suggest that these characteristics are reminiscent of SIV infection in natural disease resistant hosts.

## Additional file

**Additional file 1: Figure S1.** Effect of age and SIV-infection on weight. In comparison to mock-infected macaques, SIV-infection of aged Chinese rhesus macaques does not impact weight. Animals were weighed on a weekly basis. Median longitudinal weight (kg) over the course of infection for each group (A). Change in median weight (kg) from baseline in SIV-infected (red) and mock-infected (blue) macaques during the acute phase of infection (B). Both SIV-infected and mock infected macaques lost weight at 2 and 3 wpi. At 4 wpi, SIV-infected macaques lost a small amount of mass while mock-infected macaques slightly gained mass. Change in median weight (kg) from baseline in SIV-infected (red) and mock-infected (blue) macaques at time of necropsy (C). Mock-infected macaques gained over 1 kg by the end of the study whereas SIV-infected macaques were similar to their starting weight. **Figure S2.** Effect of age, SIV-infection and cART on body temperature. There were no changes in body temperature after SIV-infection in aged Chinese rhesus macaques. Body temperature was recorded at least once a day and the median temperature determined in two week intervals. Baseline temperature (week 0 post-infection) was the average of 12 weeks of preinoculation measurements. Time course of body temperature in SIV-infected macaques and controls (A). Lines represent median values. Time course of change in temperature (Δ °C) from baseline (B). Change in temperature from baseline during the first 4 weeks post-infection (C). Change in temperature from baseline at necropsy (D). **Figure S3.** Effect of age, SIV-infection and cART on platelet counts. Platelet counts are not significantly altered during acute SIV infection in aged rhesus macaques of Chinese origin. Time course of median platelet counts during the duration of infection (A). Lines represent median values. Platelet counts of SIV-infected and mock-infected macaques during the first 4 weeks post-infection (B). **Figure S4.** Total cell counts during the course of SIV infection. Total CD4+ T-cell counts decrease during SIV infection in aged rhesus macaques of Chinese origin. Total CD8+ T-cell and NK-cell counts shown an elevation during acute infection then fall to similar levels as mock-infected macaques. Time course change in CD4+ T-cell (A), CD8+ T-cell (B), NK-cell (C), and B-cell counts (D) during SIV and mock infection. Lines represent median values. **Figure S5.** Total monocyte subset counts during the course of SIV infection. CD14+CD16- (A), CD14+CD16+ (B), CD14-CD16+ (C) monocyte subset counts during SIV infection in aged rhesus macaques of Chinese origin. Lines represent median values. **Figure S6.** Plasma and CSF viral load versus temperature in individual SIV-infected macaques. Each graph shows the time course of plasma SIV loads, CSF SIV loads and temperature in an individual aged Chinese rhesus macaque during SIV infection. Graphs of SIV-infected macaques that did not receive treatment (Group 1) are shown in (a) and graphs of SIV-infected macaques that were treated with CART (Group 4) are shown in (b). The green shaded area represents the period macaques received CART or saline. **Figure S7.** Plasma and CSF viral load versus CD4+ and CD8+ T-cell count in SIV-infected macaques. Each graph shows the time course of plasma SIV loads, CSF SIV loads, CD4+ T-cell counts, and CD8+ T-cell counts in an individual aged Chinese rhesus macaque during SIV infection. Graphs of SIV-infected macaques that did not receive treatment (Group 1) are shown in (a) and graphs of SIV-infected macaques that were treated with CART (Group 4) are shown in (b). The green shaded area represents the period macaques received CART or saline. **Figure S8.** Plasma and CSF viral load versus reaction time and accuracy in SIV-infected macaques. Each graph shows the time course of plasma SIV loads, CSF SIV loads, reaction time (RT), and percent accuracy (Acc) in an individual aged Chinese rhesus macaque during SIV infection. Graphs of SIV-infected macaques that did not receive treatment (Group 1) are shown in (a) and graphs of SIV-infected macaques that were treated with CART (Group 4) are shown in (b). The green shaded area represents the period macaques received CART or saline. **Figure S9.** Time and accuracy in mock-infected macaques. Each graph shows the reaction time (RT) and percent accuracy (Acc) in a speeded motor task for an individual aged Chinese rhesus macaque over the study period. Graphs of mock-infected macaques that did not receive treatment (Group 2) are shown in (a) and graphs of mock-infected macaques that were treated with CART (Group 3) are shown in (b). The green shaded area represents the period macaques received CART or saline.

## Abbreviations

AIDS: acquired immune deficiency syndrome
CART: combined antiretroviral therapy
CNS: central nervous system
CSF: cerebrospinal fluid
EDTA: ethylenediaminetetraacetic acid
EIA/RIA: enzyme immunoassay/radioimmunoassay
ELISA: enzyme-linked immunosorbent assay
FTC: emtricitabine
HAD: HIV-associated dementia
HAND: HIV-associated neurocognitive disorders
HIV: human immunodeficiency virus
ISG: interferon-stimulated genes
ISH: in situ hybridization
NHP: nonhuman primate
OD: optical density
rgp130: recombinant glycoprotein 130
RM-Ch: rhesus macaques of Chinese origin
sCD14: soluble CD14
SIV: simian immunodeficiency virus
SIVmac: SIV from macaques
TDF: Tenofovir disoproxil
TMB: tetramethylbenzidine
wpi: week(s) post-infection
