# Supplementary material for: Aged Chinese-origin rhesus macaques infected with SIV develop marked viremia in absence of clinical disease, inflammation or cognitive impairment
Source: Retrovirology. 2018 Feb 1;15:17. doi: 10.1186/s12977-018-0400-y (PMC5796498; doi:10.1186/s12977-018-0400-y)
Supplement: Supplementary file 1 — Additional file 1: Figure S1. Effect of age and SIV-infection on weight. In comparison to mock-infected macaques, SIV-infection of aged Chinese rhesus macaques does not impact weight. Animals were weighed on a weekly basis. Median longitudinal weight (kg) over the course of infection for each group (A). Change in median weight (kg) from baseline in SIV-infected (red) and mock-infected (blue) macaques during the acute phase of infection (B). Both SIV-infected and mock infected macaques lost weight at 2 and 3 wpi. At 4 wpi, SIV-infected macaques lost a small amount of mass while mock-infected macaques slightly gained mass. Change in median weight (kg) from baseline in SIV-infected (red) and mock-infected (blue) macaques at time of necropsy (C). Mock-infected macaques gained over 1 kg by the end of the study whereas SIV-infected macaques were similar to their starting weight. Figure S2. Effect of age, SIV-infection and cART on body temperature. There were no changes in body temperature after SIV-infection in aged Chinese rhesus macaques. Body temperature was recorded at least once a day and the median temperature determined in two week intervals. Baseline temperature (week 0 post-infection) was the average of 12 weeks of preinoculation measurements. Time course of body temperature in SIV-infected macaques and controls (A). Lines represent median values. Time course of change in temperature (Δ °C) from baseline (B). Change in temperature from baseline during the first 4 weeks post-infection (C). Change in temperature from baseline at necropsy (D). Figure S3. Effect of age, SIV-infection and cART on platelet counts. Platelet counts are not significantly altered during acute SIV infection in aged rhesus macaques of Chinese origin. Time course of median platelet counts during the duration of infection (A). Lines represent median values. Platelet counts of SIV-infected and mock-infected macaques during the first 4 weeks post-infection (B). Figure S4. Total cell counts durin [file 12977_2018_400_MOESM1_ESM.pdf]

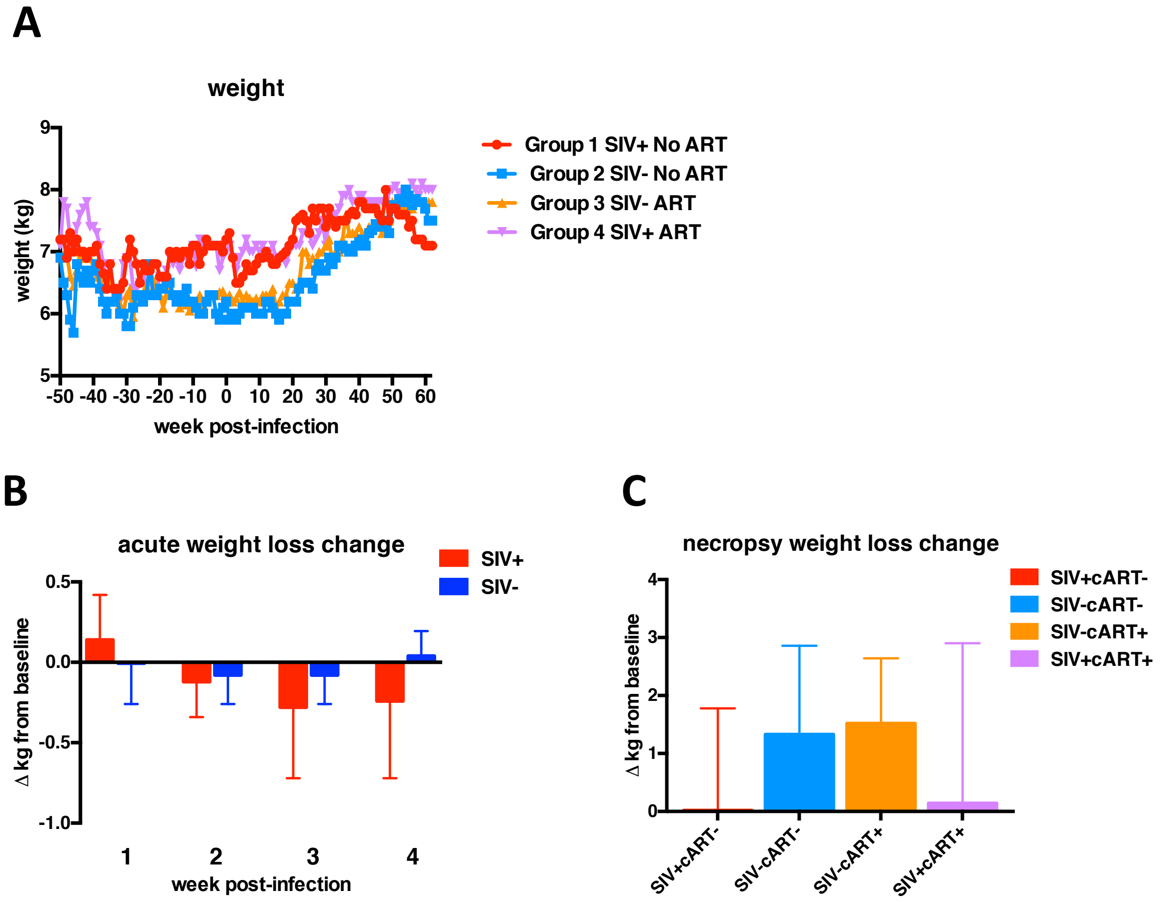

Figure S1. Effect of Age and SIV-infection on weight. In comparison to mock-infected macaques, SIV-infection of aged Chinese rhesus macaques does not impact weight. Animals were weighed on a weekly basis. Median longitudinal weight (kg) over the course of infection for each group **(A)**. Change in median weight (kg) from baseline in SIV-infected (red) and mock-infected (blue) macaques during the acute phase of infection **(B)**. Both SIV-infected and mock infected macaques lost weight at 2 and 3 wpi. At 4 wpi, SIV-infected macaques lost a small amount of mass while mock-infected macaques slightly gained mass. Change in median weight (kg) from baseline in SIV-infected (red) and mock-infected (blue) macaques at time of necropsy **(C)**. Mock-infected macaques gained over 1 kg by the end of the study whereas SIV-infected macaques were similar to their starting weight.

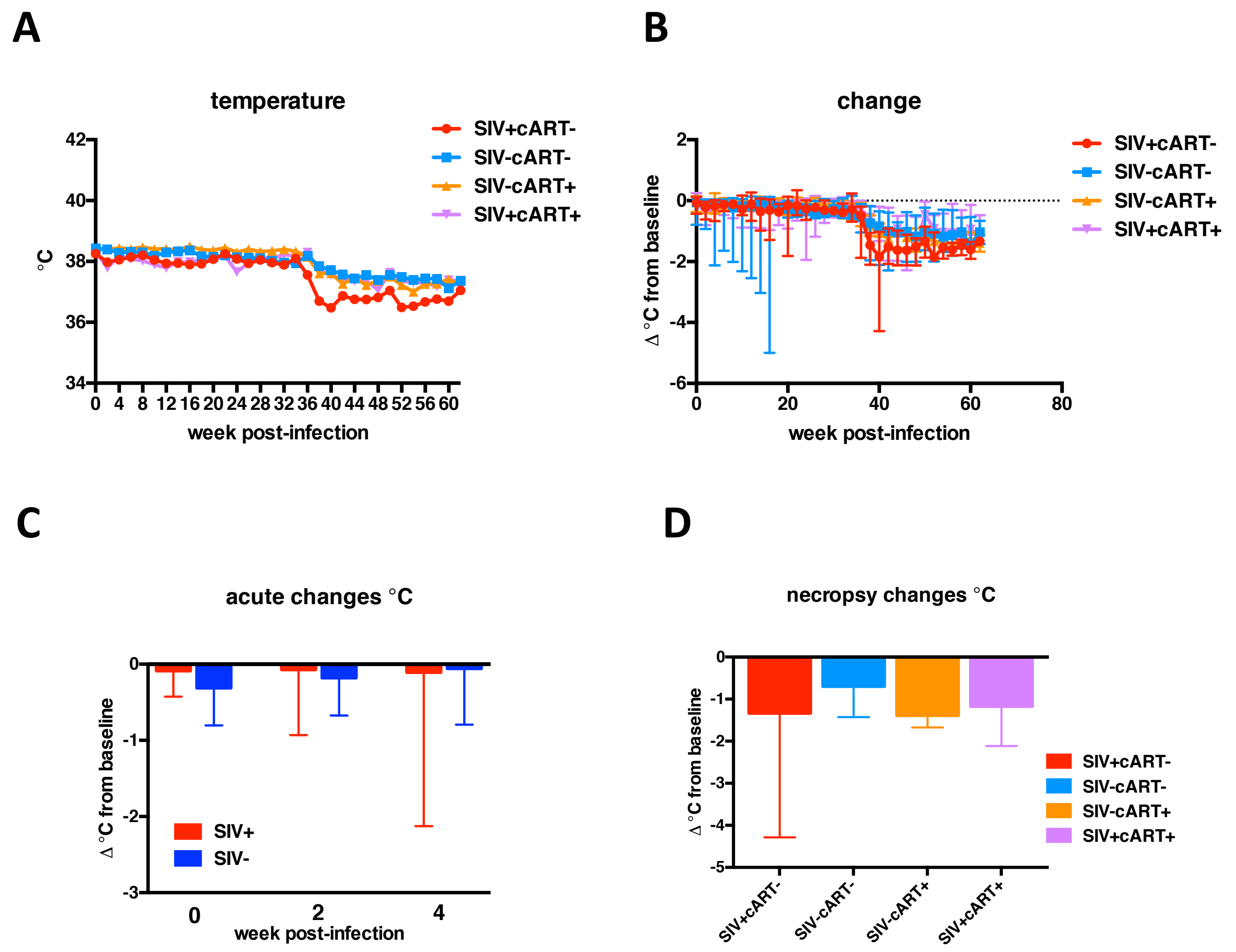

Figure S2. Effect of Age, SIV-infection and cART on body temperature. There were no changes in body temperature after SIV-infection in aged Chinese rhesus macaques. Body temperature was recorded at least once a day and the median temperature determined in two week intervals. Baseline temperature (week 0 post-infection) was the average of 12 weeks of preinoculation measurements. Time course of body temperature in SIV-infected macaques and controls **(A)**. Lines represent median values. Time course of change in temperature (Δ °C) from baseline **(B)**. Change in temperature from baseline during the first 4 weeks post-infection **(C)**. Change in temperature from baseline at necropsy **(D)**.

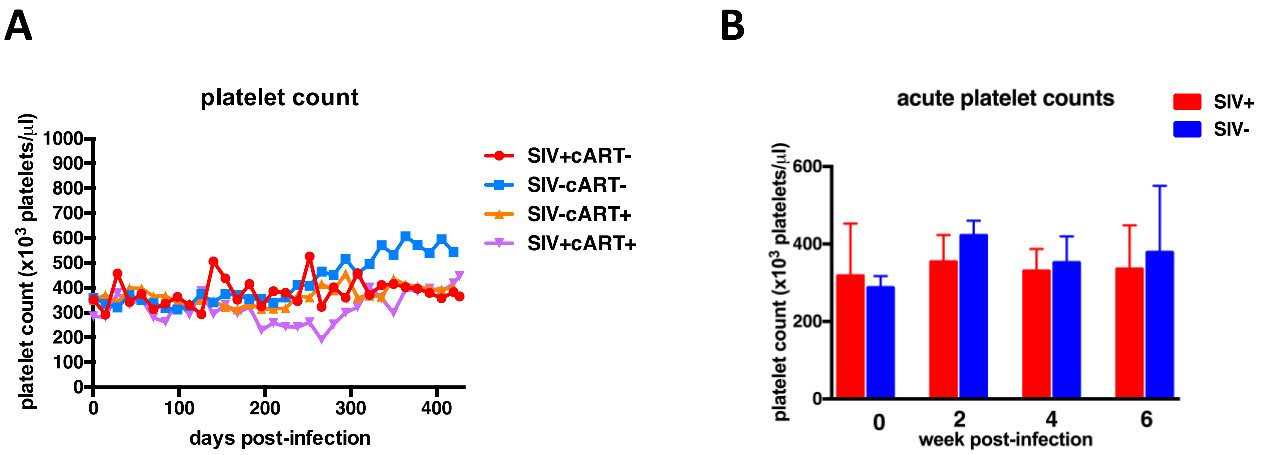

Figure S3. Effect of Age, SIV-infection and cART on platelet counts. Platelet counts are not significantly altered during acute SIV infection in aged rhesus macaques of Chinese origin. Time course of median platelet counts during the duration of infection **(A)**. Lines represent median values. Platelet counts of SIV-infected and mock-infected macaques during the first 4 weeks post-infection **(B)**.

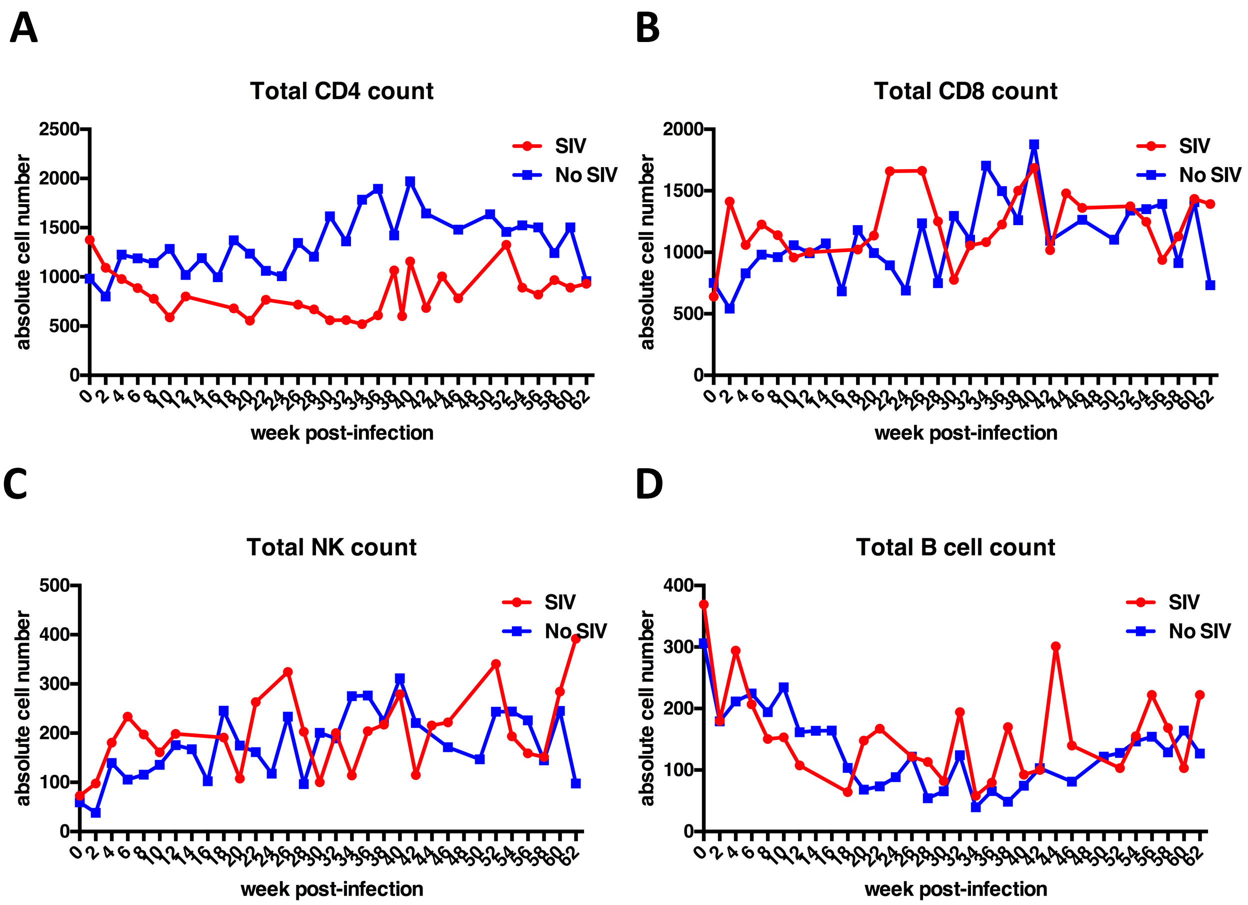

Figure S4. Total cell counts during the course of SIV infection. Total CD4+ T-cell counts decrease during SIV infection in aged rhesus macaques of Chinese origin. Total CD8+ T-cell and NK-cell counts shown an elevation during acute infection then fall to similar levels as mock-infected macaques. Time course change in CD4+ T-cell **(A)**, CD8+ T-cell **(B)**, NK-cell **(C)**, and B-cell counts **(D)** during SIV and mock infection. Lines represent median values.

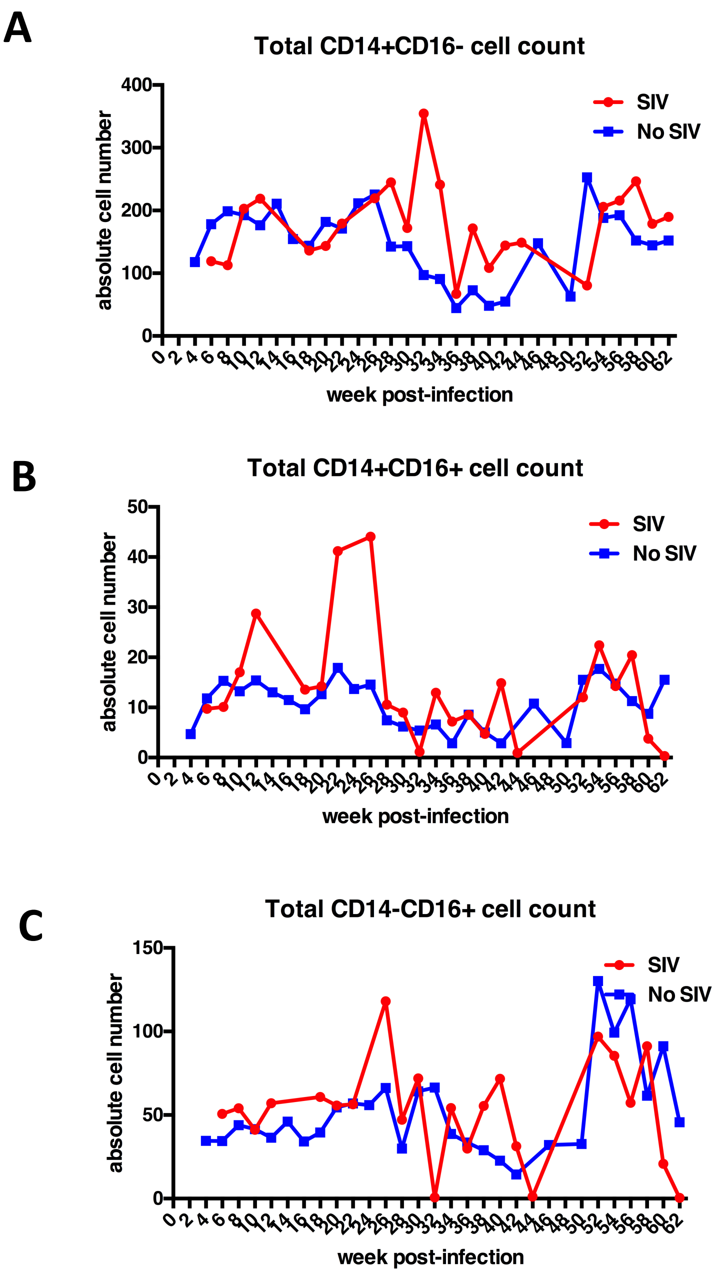

Figure S5. Total monocyte subset counts during the course of SIV infection. CD14+CD16- **(A)**, CD14+CD16+ **(B)**, CD14-CD16+ **(C)** monocyte subset counts during SIV infection in aged rhesus macaques of Chinese origin. Lines represent median values.

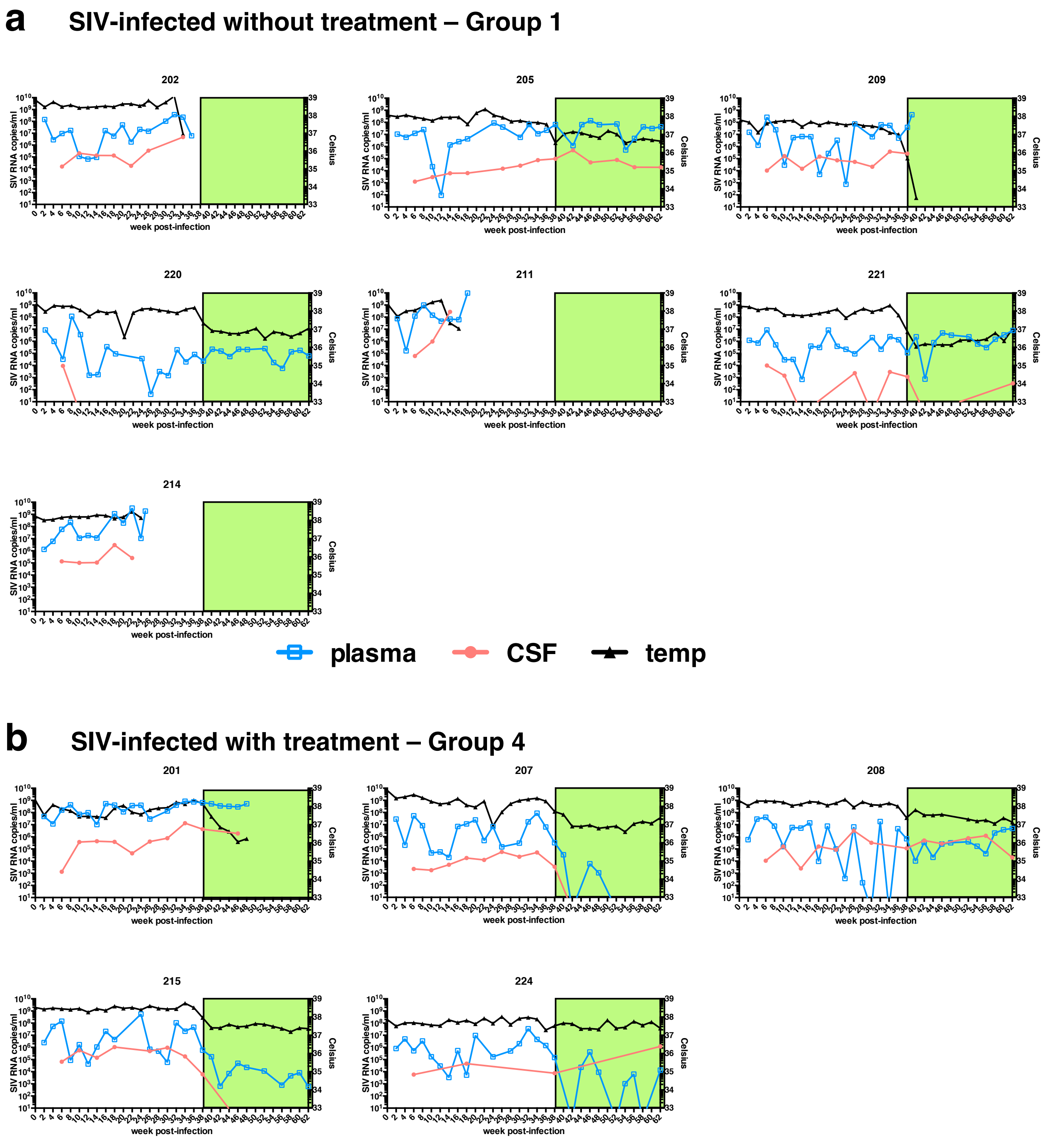

Figure S6. Plasma and CSF viral load versus temperature in individual SIV-infected macaques. Each graph shows the time course of plasma SIV loads, CSF SIV loads and temperature in an individual aged Chinese rhesus macaque during SIV infection. Graphs of SIV-infected macaques that did not receive treatment (Group 1) are shown in **(a)** and graphs of SIV-infected macaques that were treated with CART (Group 4) are shown in **(b)**. The green shaded area represents the period macaques received CART or saline.

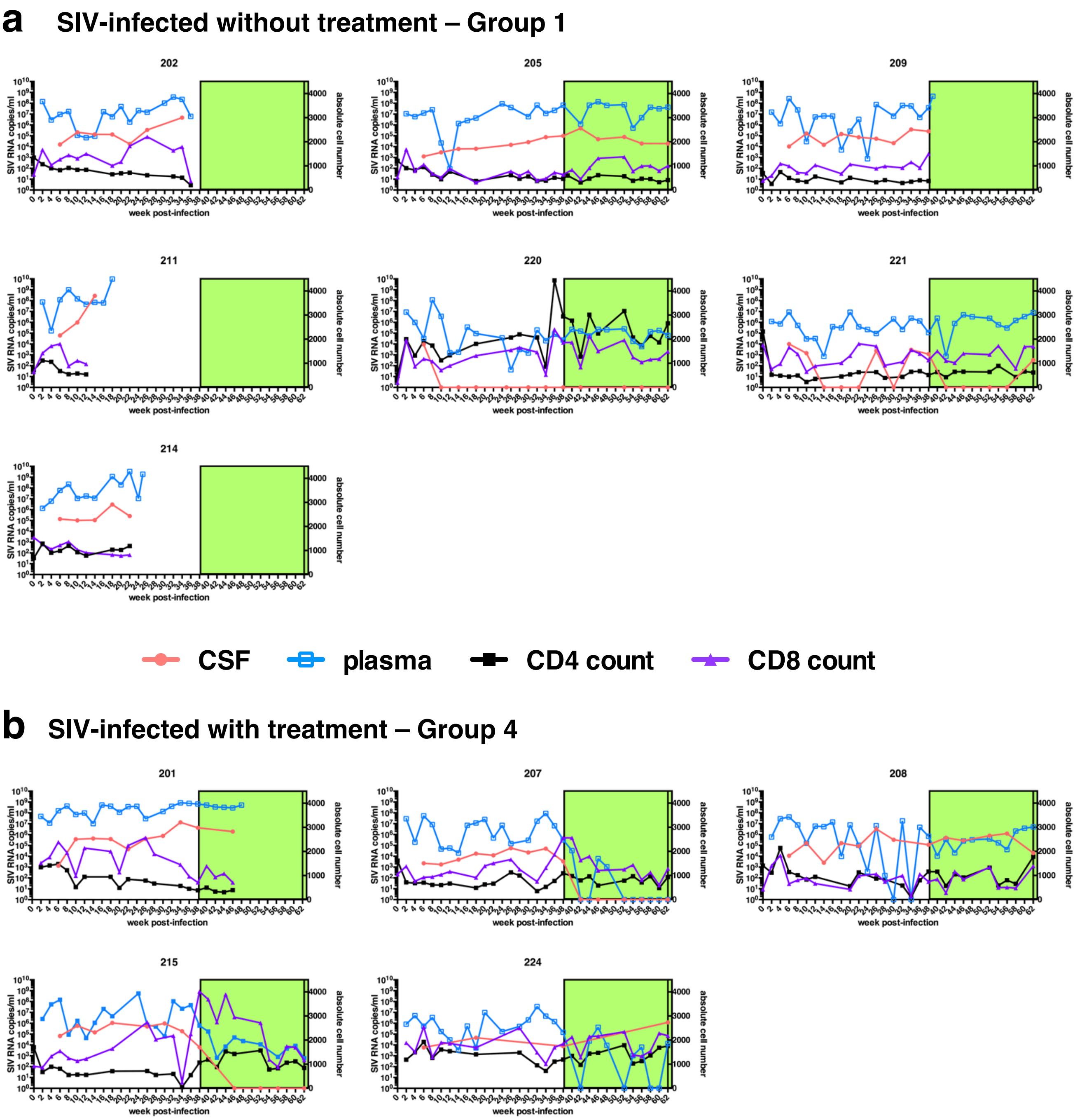

Figure S7. Plasma and CSF viral load versus CD4+ and CD8+ T-cell count in SIV-infected macaques. Each graph shows the time course of plasma SIV loads, CSF SIV loads, CD4+ T-cell counts, and CD8+ T-cell counts in an individual aged Chinese rhesus macaque during SIV infection. Graphs of SIV-infected macaques that did not receive treatment (Group 1) are shown in **(a)** and graphs of SIV-infected macaques that were treated with CART (Group 4) are shown in **(b)**. The green shaded area represents the period macaques received CART or saline.

a **SIV-infected without treatment – Group 1**

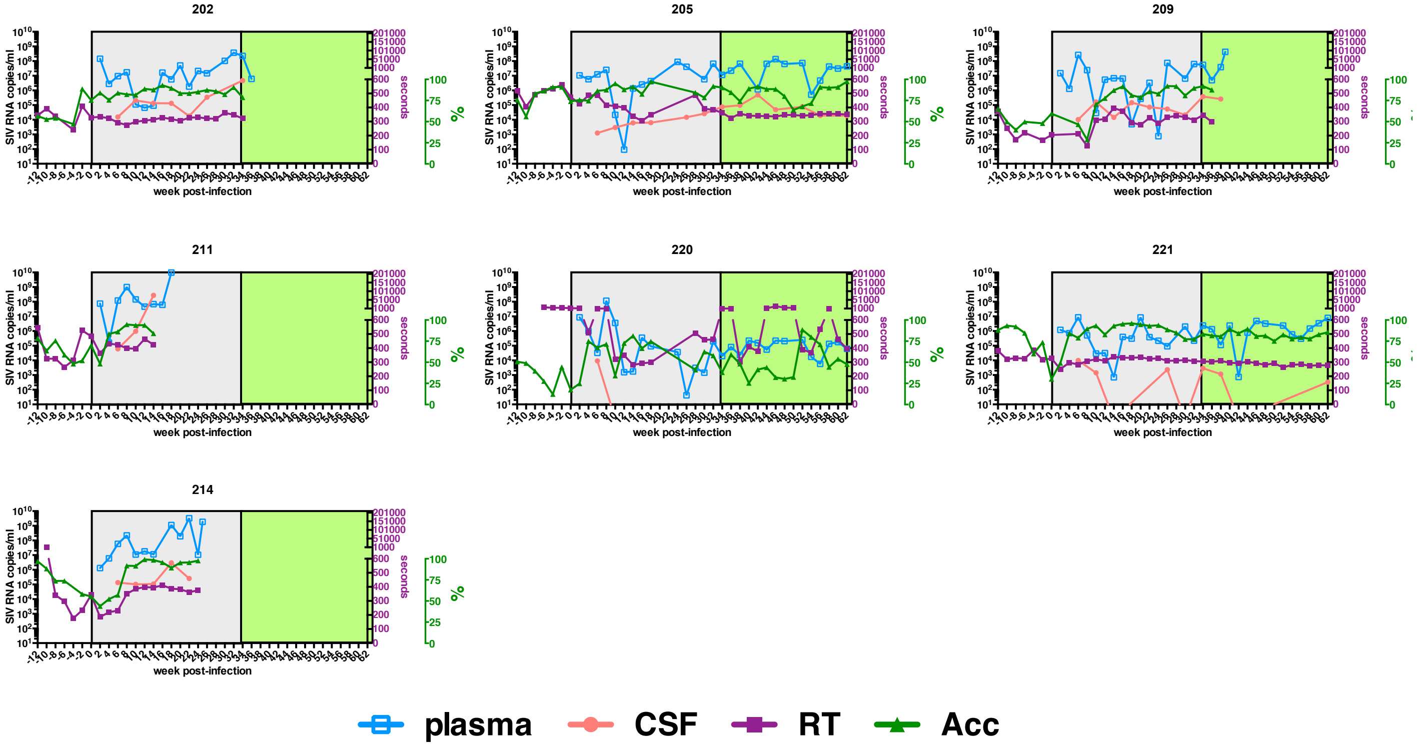

b **SIV-infected with treatment – Group 4**

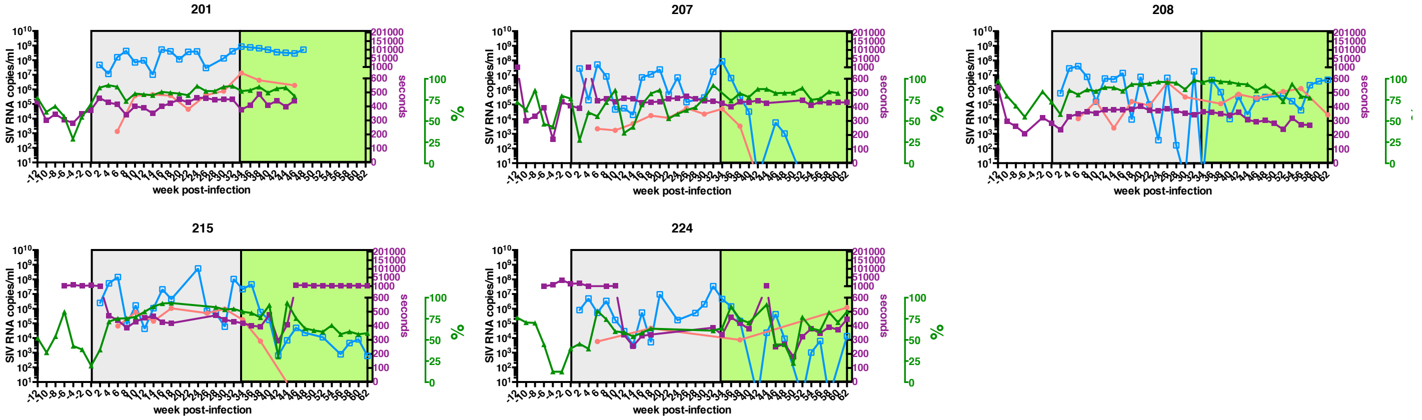

Figure S8. Plasma and CSF viral load versus reaction time and accuracy in SIV-infected macaques. Each graph shows the time course of plasma SIV loads, CSF SIV loads, reaction time (RT), and percent accuracy (Acc) in an individual aged Chinese rhesus macaque during SIV infection. Graphs of SIV-infected macaques that did not receive treatment (Group 1) are shown in **(a)** and graphs of SIV-infected macaques that were treated with CART (Group 4) are shown in **(b)**. The green shaded area represents the period macaques received CART or saline.

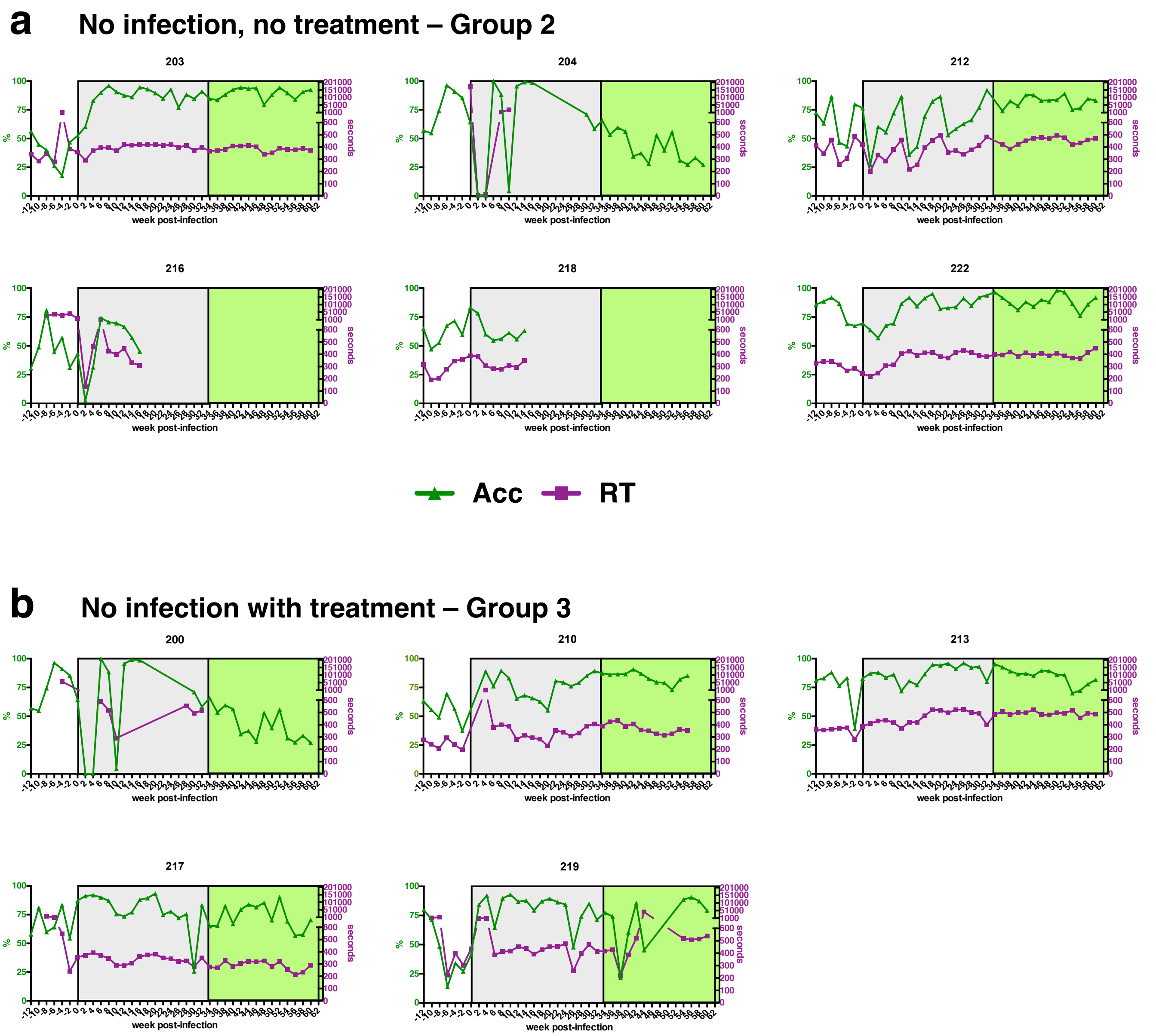

Figure S9. Time and accuracy in mock-infected macaques. Each graph shows the reaction time (RT) and percent accuracy (Acc) in a speeded motor task for an individual aged Chinese rhesus macaque over the study period. Graphs of mock-infected macaques that did not receive treatment (Group 2) are shown in **(a)** and graphs of mock-infected macaques that were treated with CART (Group 3) are shown in **(b)**. The green shaded area represents the period macaques received CART or saline.
